# Austin District Medical Society

**Published:** 1891-01

**Authors:** 


### AUSTIN DISTRICT MEDICAL SOCIETY.

The thirteenth quarterly meeting of the Austin District Medical Society was held in Medical Hall, December 19, 1890, with the largest attendance since the organization over three years ago. About sixty physicians were present, and a more enjoyable time, scientifically and socially, the society never had. Dr. R. R. Walker, first physician to the Lunatic Asylum at Austin, and Dr. Ault, of Lampasas, were admitted to membership.

There were eight excellent papers read which in due time will appear in the JOURNAL.

Dr. A. Garwood, of New Braunfels, reported the death of Dr. G. B. Underhill. See memorial elsewhere in this JOURNAL.

The following officers were elected for the ensuing year: Dr. J. W. McLaughlin, President; Dr. W. A. Ellison, 1st Vice-President; Dr. J. W. Hamilton, 2nd Vice-President; Dr. T. J. Bennett, Secretary and Treasurer, holds over one year; Drs. A. N. Denton, A. J. Davis and F. R. Martin, Censors.

It affords a great deal of pleasure to refer to the Austin District Medical Society as a grand success. Such it is. And it is commended to all reputable physicians accessible to Austin. Gentlemen, you cannot do without the strong arm of a good working society around you if you want to progress in your profession.

The proceedings of the day closed with a banquet at night, after which all went in a body to the opera house and witnessed an excellent play.

